# Neurogenic Stunned Myocardium: A Narrative Review of Cardiopulmonary Failure Related to Subarachnoidal Haemorrhage

**DOI:** 10.7759/cureus.93133

**Published:** 2025-09-24

**Authors:** Dharmanand Ramnarain, Susan Deenen, Laura Boerboom, Jos van Oers, Sjaak Pouwels

**Affiliations:** 1 Intensive Care Medicine, Elisabeth-Tweesteden Hospital, Tilburg, NLD; 2 Intensive Care Medicine, Elisabeth Tweesteden Hospital, Tilburg, NLD; 3 Medical Informatics, Elisabeth Tweesteden Hospital, Tilburg, NLD

**Keywords:** cardiomyopathy, intensive care, neuro icu, subarachnoid bleeding, subarachnoid bleeding (sab), takotsubo cardiomyopathy

## Abstract

Acute subarachnoid haemorrhage (SAH) is an acute bleeding in the subarachnoid space in the brain. Due to various pathophysiological sequelae, SAH can be accompanied by acute cardiac failure. Patients who suffer from cardiac failure due to SAH have a high morbidity and mortality. Although the pathophysiology is not completely understood in both clinical and experimental studies, several attempts have been made and published to explain the pathophysiological mechanisms of cardiac failure in SAH patients. Two forms of cardiac failure are described after neurologic injury (like SAH) which are neurogenic stunned myocardium (NSM) with or without diffuse and/or regional wall motion abnormalities (RWMA), and a more specific form called takotsubo cardiomyopathy. This review aims to summarise the current literature on cardiac abnormalities and related pathophysiology in patients with SAH.

## Introduction and background

Cardiac failure can occur following acute subarachnoid haemorrhage (SAH) and is responsible for significant morbidity and mortality [1-4]. Although not fully understood, several research studies (both clinical and experimental) have been published trying to explain the pathophysiological mechanism of cardiac failure in these patients.

In the literature, mainly two forms of cardiac failure are described after neurologic injury (like SAH): neurogenic stunned myocardium (NSM) with or without diffuse and/or regional wall motion abnormalities (RWMAs), and a more specific form called takotsubo cardiomyopathy. In 2018, it was named takotsubo syndrome (TTS) because of the reversible character of this disease. This makes it distinct from known cardiomyopathies. Secondly, the suggested change also reflects the common pathophysiology with ischaemic syndromes. Therefore, the term “cardiomyopathy” was deemed not suitable anymore [5]. 'Takotsubo' refers to the morphological shape of the left ventricle, typically looking like a Japanese octopus trap [6].

This syndrome was first described in SAH patients by Burch et al. in the 1950s as strain on the heart after acute SAH [7]. This was the first report on SAH-related cardiac failure. They reported electrocardiographic (ECG) abnormalities similar to those seen in acute coronary syndromes [7]. However, the first report on takotsubo cardiomyopathy was described in the 1990s by Sato et al. [7]. With the introduction of echocardiography, left ventricular wall motion abnormalities (LVWMA) could be visualised at an early phase of the disease. In both forms (NSM and TTS), specific features can be seen on echocardiographic examination. Some authors have argued that both forms of cardiomyopathy are the same and that we should stop making a distinction [8-10]. Several echocardiographic studies have indeed shown that both forms are alike when looking at specific echocardiographic characteristics [8-10]. In a study by Ancona et al., in a cohort of 36 SAH patients, 22 of them were diagnosed with TTS (diagnosed based on Reverse Mayo Clinic criteria), and 14 patients in this cohort were diagnosed as having NSM with transient RWMA, acute ischaemic changes on ECG with low levels of cardiac troponin [11]. When comparing echocardiographic results in both TSS and NSM, typical apical ballooning and midventricular and/or basal ventricular wall motion abnormalities were seen. There is no consensus on the best definition, and therefore, the aim of this study was to summarise current literature on SAH-related cardiac failure, whether TTS or NSM. 

This review aims to summarise the current literature on acute neurologic disease-related cardiac abnormalities and related pathophysiology in patients with SAH. We will use the term NSM, as both TTS and NSM are considered the same entity with the same pathophysiologic mechanism, as we will describe in the review.

## Review

Epidemiology

The true incidence of SAH-related NSM is difficult to find in the literature. In a systematic review, the incidence of NSM varied from 0.8% to 33% [12]. In a cross-sectional study of all admitted neurology patients registered from 2006 to 2014 from the National Inpatient Sample, the odds of developing NSM were the highest in patients with SAH (OR 11.7; 95% CI 10.2-13.4), followed by status epilepticus (OR 4.9; 95% CI 3.7-6.3) and seizures (OR 1.3; 95% CI 1.1-1.5). In this cohort, female gender was strongly associated with NSM (OR 5.1; CI 4.9-5.4) [13]. In a study of 713 SAH patients receiving daily echocardiography, 28% of these patients developed NSM-related RWMA. In total, 15% of these patients developed global systolic dysfunction with a left ventricular ejection fraction (LVEF)​​ less than 50% [14]. Others found the same 28% of NSM-related RWMA, and 11% had LVEF < 50% [15].

In a recent study by Kim et al., it was shown that after analysis of 14 studies including 2,234 patients, echocardiographic evidence of NSM-related RWMA was related to a significant increase in in-hospital mortality (OR 2.37; 95% CI 1.74-3.25 and 2.82; 95% CI 1.2-6.6), respectively [15-23].

Female gender, older age (>60 years), more severe bleeding on CT scan, and ruptured aneurysms of the posterior circulation are more associated with the development of NSM than anterior circulation aneurysms [16-18]. The prognosis of NSM in SAH patients is mainly determined by the development of complications of SAH (rebleeding, hydrocephalus, vasospasm, secondary ischaemia) [3, 19, 20]. Poor neurological outcome has also been described in patients with cardiac arrest, right ventricular wall motion abnormalities, and the need for inotropic support [20, 21]. In a large series of 800 patients with SAH, mortality was 22% in patients with NSM compared to 15% in patients with uncomplicated SAH [19]. Others reported an in-hospital mortality of even 29.8% [22]. 

Although NSM is a self-limiting condition, cardiogenic shock and even death can occur, comparable to patients with acute coronary syndromes, especially in the acute phase, and are seen in about 20% of all NSM patients [24]. In a study by Bihorac et al., the long-term outcome of SAH patients who developed NSM was investigated in 715 SAH patients. In 200 patients (28%), an echocardiography was performed because of clinical evidence of cardiac failure [25]. NSM was detected in 59 (8%) patients, with 34 showing apical ballooning and 25 showing basal wall motion abnormalities. In-hospital mortality of SAH patients with NSM was 15% and not significantly different from that of patients without NSM. After a median follow-up of seven years, adjusted survival in these 200 patients was significantly decreased compared with patients without any indication for echocardiography (Cox regression analysis; hazard ratio (HR) 1.70, 95% CI 1.13-2.54). In patients with proven NSM, there was no significant increase in survival rate (HR 1.43, 95% CI: 0.75-2.59). Patients with proven NSM had a higher OR for severe sepsis (OR 2.7, 95% CI 1.04-7.3) and even higher in patients with basal-type wall motion abnormalities (OR 4.8, 95% CI 1.4-16.3). Although no causal inference can be concluded based on the results of this study, it could suggest that early detection of possible NSM could have prognostic implications. Further studies are warranted on early echocardiography and outcome in SAH patients [25].

Diagnostic criteria of NSM

NSM can vary widely in LVWMA, which extends beyond the distribution area of a single coronary artery. Right ventricular wall motion abnormalities can also be part of NSM. Four types of NSM have been described in the literature: 1) the most common type describes apical ballooning [26, 27]; 2) midventricular wall motion abnormalities [28-30]; 3) basal wall motion abnormalities [24, 31]; and 4) focal wall motion abnormalities [24, 32]. Due to the heterogeneity of NSM, there is still no universal consensus on diagnostic criteria, and for NSM, no diagnostic criteria exist. Recently, the European Society of Cardiology developed new diagnostic criteria using the term TTS [33]. In these criteria, neurological diseases are recognised as one of the primary criteria for TTS. Also, coronary artery disease can coexist in patients having TTS, and it's not an exclusion criterion anymore, in contrast to other well-known criteria, such as the Revised Mayo Clinic Diagnostic criteria [34]. InterTAK Diagnostic Criteria are presented in Table 1. In 2019, the Heart Failure Association also updated their criteria, in which recovery of ventricular systolic function after three to six months was mandatory [35]. 

**Table 1:** InterTAK Diagnostic Criteria *Table created by the authors using information from references [33–35]. ^a^ Wall motion abnormalities may remain for a prolonged period of time, or documentation of recovery may not be possible; for example, death occurs before evidence of recovery is captured. ^b^ Cardiac magnetic resonance imaging is recommended to exclude infectious myocarditis and to confirm the diagnosis of takotsubo syndrome.

InterTAK Diagnostic Criteria
Patients show transient^a^ left ventricular dysfunction (hypokinesia, akinesia, or dyskinesia) presenting as apical ballooning or midventricular, basal, or focal wall motion abnormalities. Right ventricular involvement can be present. Besides these regional wall motion patterns, transitions between all types can exist. The regional wall motion abnormality usually extends beyond a single epicardial vascular distribution; however, rare cases can exist where the regional wall motion abnormality is present in the subtended myocardial territory of a single coronary artery (focal TTS)^b ^
An emotional, physical, or combined trigger can precede the takotsubo syndrome event, but this is not obligatory.
Neurologic disorders (e.g. subarachnoid haemorrhage, stroke/transient ischemic attack, or seizures) as well as pheochromocytoma may serve as triggers for takotsubo syndrome.
New ECG abnormalities are present (ST-segment elevation, ST-segment depression, T-wave inversion, and QTc prolongation); however, rare cases exist without any ECG changes.
Levels of cardiac biomarkers (troponin and creatine kinase) are moderately elevated in most cases; significant elevation of brain natriuretic peptide is common.
Significant coronary artery disease is not a contradiction in takotsubo syndrome.
Patients have no evidence of infectious myocarditis^b^.
Postmenopausal women are predominantly affected.
Systolic function recovery within three to six months

Echocardiographic LVWMAs are the key findings in these patients, together with or without elevated levels of troponin and ECG abnormalities [36]. In NSM, ECG abnormalities can vary widely, but some ECG screening tools for diagnosing NSM have been proposed with good sensitivity and specificity.

Echocardiography is essential for visualising ventricular wall motion abnormalities of the left as well as the right ventricle. It is an important tool for follow-up of heart failure by screening for complications such as ventricular wall rupture, mitral valve regurgitation, pericardial effusion, left ventricle outflow problems, involvement of the right ventricle, and thrombus formation [37, 38]. Table 2 shows the criteria for diagnosing TTS or NSM [28, 39, 40]. 

**Table 2:** Echocardiographic variants of left ventricular (LV) regional wall motion abnormalities (RWMA) as described by the International Expert Consensus Document on takotsubo syndrome (TTS) *Table created by the authors using information from references [33–35]. ACS: acute coronary syndrome

Echocardiographic variants of LV RWMA as described by the International Expert Consensus Document on TTS
Apical ballooning, hypo-, a-, or dyskinesia of mid-apical myocardial segments is typical, sometimes associated with hypokinetic midsegments. The anterior or entire interventricular septum, the inferior midventricular anterolateral wall may also be involved. LV twisting on 2D speckle-tracking imaging is reduced or reversed to clockwise apical rotation, and the rate of untwisting (a sensitive index of regional diastolic dysfunction) is reduced in the acute phase
Midventricular TTS is featured by hypo-, a-, or dyskinesia of midventricular segments, most often resembling a cuff.
Basal forms where only basal segments are involved. This phenotype is rare and appears commonly in patients with subarachnoid haemorrhage, epinephrine-induced TTS or phaeochromocytoma.
Focal TTS, mostly involving an anterolateral segment, has been described. Differentiating this unusual TTS type from ACS or myocarditis requires cardiac magnetic resonance imaging.

Regarding echocardiographic studies in SAH patients, more authors address the overall left ventricular dysfunction (including RWMA, NSM, TTS, and decreased EF) instead of making specific differences in TTS and NSM [14, 25, 41-48]. Several authors reported poor functional outcome, increased mortality, and left ventricular dysfunction (LVD) in SAH patients [1-3]. The conclusion of a recent meta-analysis was that addressing all LVD as RWMA related to NSM could provide better prognostic information of in-hospital mortality [23].

Pathophysiology of NSM

**Figure 1:**
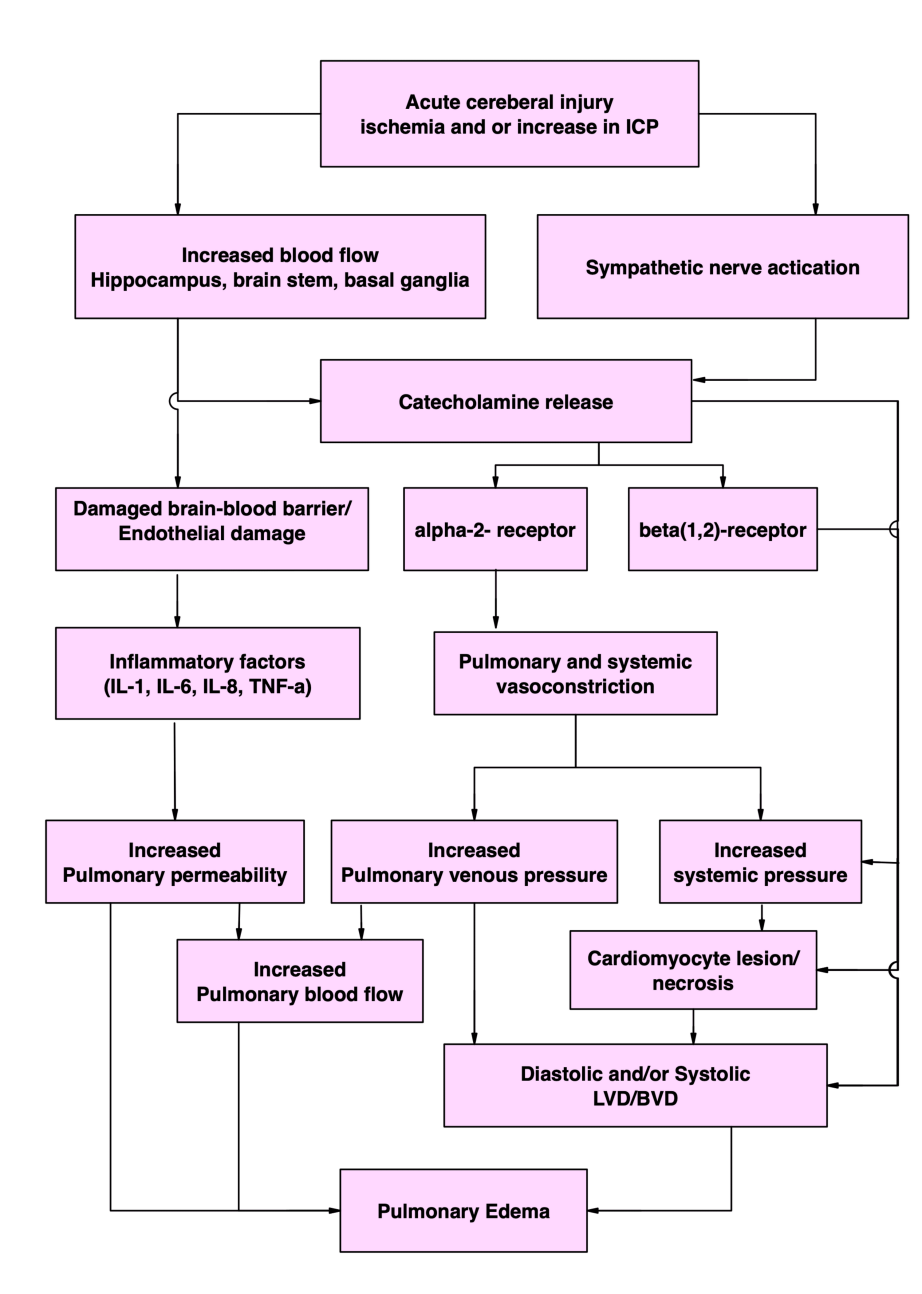
Overview of the pathophysiological mechanisms related to the development of neurogenic stunned myocardium in patients with subarachnoid haemorrhage (SAH). ICP: intracranial pressure; LVD: left ventricular dysfunction; BVD: bi-ventricular dysfunction; IL: interleukin; TNF: tumour necrosis factor The figure has been created by author D. Ramnarain.

Figure 1 shows an overview of the pathophysiological mechanisms related to the development of NSM in patients with SAH.

Activation of the sympathetic nervous system

The exact pathophysiology is not well understood, but activation of the sympathetic nervous system seems to be the major cause in the early stage of NSM. In response to stress, catecholamines are released by activation of the hippocampus, brainstem and basal ganglia [49-52]. High concentrations of catecholamines are also found in patients with pheochromocytoma [53] and central nervous system diseases [54, 55].

Suzuki et al. demonstrated that specific activation of cerebral regions was associated with NSM [50]. They showed that during the acute phase of NSM, the cerebral blood flow significantly increased in the hippocampus, brainstem and basal ganglia and decreased in the prefrontal cortex. Later in the course of the disease, wall motion abnormalities normalised, but blood flow was still increased in specific regions of the brain, suggesting a long-lasting psychological stress in patients with NSM [56].

Multivessel epicardial spasms

NSM has been associated with endothelial dysfunction. Endothelium-dependent dilatation is reduced after emotional stress [57]. NSM patients have impaired flow-mediated dilatation compared to healthy controls, which gradually improves after several weeks [58]. In some patients with NSM, a predisposition to coronary vasospasm has been reported [59]. Endothelial dysfunction has been associated with oxidative stress. A recent animal study has found that hydrogen sulphide relieved cardiac dysfunction by decreasing oxidative stress [60]. Nanno et al. measured 8-hydroxy-2’-deoxyguanosine (8-OHdG) and norepinephrine levels as markers of oxidative stress in NSM patients and patients with myocardial infarction. In these patients with NSM, higher levels of norepinephrine were found, and 8-OHdG levels changed significantly with changes in wall motion scores in the course of TTS [61]. 

Microcirculatory dysfunction

Coronary microvascular blood flow is reduced in the acute phase of NSM [62-64]. Alpha-1 receptors and endothelin receptor type A are predominantly present in the coronary microvasculature, which are activated by high levels of catecholamines and endothelin [65]. By analysing circulating microRNAs as a biomarker of cardiovascular disease, it was possible to differentiate different microRNAs in patients with NSM and healthy controls and patients with myocardial infarction. In patients with NSM, high endothelin-1 and low levels of endothelin-1-regulating microRNA-125-5p were found [66]. Galiuto et al. studied acute microvascular dysfunction by intravenous administration of adenosine, which showed a transient increase in myocardial perfusion, LV wall motion and LVEF in patients with NSM [26]. In addition, endomyocardial biopsies of patients with NSM have shown apoptosis of microvascular endothelial cells [67].

Catecholamine toxicity on cardiomyocytes

Catecholamines have a direct toxic effect on cardiomyocytes, as revealed by endomyocardial biopsy studies showing contraction band necrosis, hypercontracted sarcomeres, dense eosinophilic transverse bands, and interstitial mononuclear inflammation [68]. In NSM, catecholamines decrease the myocyte activity through cAMP-mediated Ca2+ overload, which eventually can lead to contractile dysfunction [69]. Accumulation of fat in cardiomyocytes occurs due to high doses of catecholamines, as shown in biopsy studies in both animal [70] and NSM patients [71]. In mammalian hearts, the beta-adrenergic receptor density is highest in the apex, while sympathetic nerve innervation is very low [72-74], suggesting that high levels of catecholamine can cause diminished coronary blood flow. High catecholamine levels can also paradoxically cause negative inotropic effects (i.e., low cardiac output) due to an activation of beta-2-receptors, which signal more to negatively inotropic pathways [75-77]. Another effect of activation of beta2-receptors is the stimulation of NO synthase, leading to negative inotropic situations and inflammation as seen in patients with NSM [78], but also in postmortem biopsies of the heart [79].

The regional difference in density of beta-2 receptors in the left ventricle could explain the pattern of ventricular dysfunction seen in different patients. Animal studies have also shown that beta-2 receptors are more expressed at the apex and less at the basal segment, while sympathetic nerves and norepinephrine receptors have the opposite distribution: more in the basal segments and less in the apex [72, 74]. Based on this evidence, differences in NSM-related LVWMA could possibly be explained. In both clinical entities, there is a catecholamine-mediated myocardial injury, but with a different pattern of receptor activation, where epinephrine induces apical dysfunction and norepinephrine induces more dysfunction in the basal segment in NSM [72, 74].

Hormonal factors

Oestrogens increase vasomotor tone by up-regulation of the NO synthase [80] and can attenuate catecholamine-mediated vasoconstriction and decrease the sympathetic nerve response to mental stress in perimenopausal women [81, 82]. In women with SAH, low levels of oestrogen have been associated with an increased risk of LV wall motion abnormalities [83]. In particular, women after 55 years (i.e., declining levels of oestrogens after menopause) have an almost five times higher risk of developing NSM compared with younger women [84].

Predisposing genetic factors

Some experts state that genetic predisposition to TTS exists, illustrated by the report of five cases of TTS in female members of the same families [85-89]. However, polymorphism in adrenergic genes can affect receptor function and downstream signalling associated with cardiac dysfunction in patients with SAH [90, 91]. Zaroff et al. [91] described beta1-adrenergic and beta2-adrenergic receptor variants in patients with SAH that were associated with greater release of troponin I. They also described an alpha2-adrenergic receptor amino deletion and reduced LVEF [91].

In a recent experimental study by Borchert et al. [92], fibroblast cells of patients with TTS and healthy controls were integrated into pluripotent stem cells and differentiated towards functional cardiomyocytes. In an experimental model, both of these ‘cell lines’ were stimulated with catecholamines. A specific beta1-receptor type was used to mediate the effect of catecholamines on B-receptor signalling. This study showed an increase in multiple cardiac stress markers. The engineered cardiomyocytes showed impaired contractility and higher sensitivity to inotropes compared to controls. Also, electric signalling was altered in these cells, and there was more lipid accumulation. Additional DNA analysis uncovered genetic variants of cardiomyopathies and cardiac arrhythmias [92].

Neurogenic pulmonary oedema (NPE)

NPE is common in patients with SAH and other central nervous system injuries, like severe head injury [93], and is a life-threatening complication. NPE prevalence reported in the literature varies between 2% and 31% [94]. Vertebral artery dissection and severe World Federation of Neurological Surgeons (WFNS) grade on admission are risk factors for NPE [94]. NPE is associated with worse outcomes and high mortality [95-97]. In a large cohort of 170 SAH patients, illness severity, amount of blood on CT scan, blood transfusion, and severe sepsis in the ICU were risk factors for NPE and increased in-hospital mortality (OR, 1.63; 95% CI, 1.03-2.57). It was also independently associated with an increased ICU length of stay (15%, 95% confidence interval, 5%-27%) [98].

There are several mechanisms involved in the development of NPE. The overactivation of the sympathetic nervous system due to catecholamine release is the same mechanism as in cardiomyopathy [99-101]. Cardiomyopathy is associated with wall motion abnormalities of the left as well as the right ventricle, which can result in both systolic dysfunction and diastolic cardiac dysfunction and therefore in NPE [102]. High intracranial pressures after SAH can cause ischaemic insults in areas of the hypothalamus and medulla oblongata, leading to massive sympathetic activation. This results in pulmonary and systemic vasoconstriction and an increase in pulmonary hydrostatic pressure, causing a fluid shift from pulmonary capillaries into the lung tissue. Another mechanism described in the literature is the inflammatory response in the brain as a result of cerebral insults. This leads to the production of brain cytokines, which can trigger processes in other organs, like the pathophysiological cascade leading to NPE. Finally, local inflammatory processes will lead to capillary leakage, leading to pulmonary oedema [102].

In the 80s and 90s, NPE was a well-known complication related to increased hydrostatic pressure due to triple-H therapy, which was given to patients with SAH with suspected vasospasm (hypertension, hypervolemia and hemodilution) with a prevalence of 14.3%-16% [103-105]. Due to the lack of benefit and potential harm of massive fluid infusion, hypervolemia therapy has no place in the ICU treatment of SAH patients nowadays. In patients with close haemodynamic monitoring and fluid-restricted management, NPE incidence could be reduced from 14% to 6% [106].

Management of NSM

Patients with SAH should be treated in a specialised hospital with a dedicated neurological intensive care unit, and aneurysm treatment should be done as soon as possible. Supportive therapy is started in order to prevent early complications and to minimise further damage caused by ongoing sympathetic stimulation of the central nervous system. Prolonged cardiac failure and pulmonary oedema are associated with increased risk of DCI and poor functional outcome [107].

Treatment in case of NSM in patients with SAH is supportive and is based on principles seen in primary cardiac failure, mainly reduction of preload, afterload and concomitant inotropic support [108]. A reduction in preload is achieved by administering diuretics, but not without risks. SAH patients are at high risk for developing hypovolemia. Hypovolemia is related to increased risk of developing vasospasm, delayed cerebral ischaemia and poor outcome [108-110].

In a study conducted by Hoff et al. [111], patients who developed pulmonary oedema after SAH had decreased circulating blood volume compared to patients who did not develop pulmonary oedema. Because hypovolemia is related to increased risk for delayed cerebral ischaemia, the benefits of the use of diuretics should be weighed against the potential harm of increased risk of hypovolemia.

Hypovolemia could result in severe hypotension and should be avoided, as a recent study by Gathier C et al. showed that a mean arterial pressure below 60 mmHg was associated with an increased risk of rebleed [112]. A mean arterial pressure below 100 mmHg was associated with decreased risk of bleeding (HR 0.30, 95% CI 0.11-0.80) in this study.

Early treatment of SAH is important and should be done in hospitals with dedicated neurosurgical, radiological and ICU staff. It is of utmost importance that SAH patients are referred to these centres as soon as possible. Treatment in specialised centres is related to higher survival rates and better clinical outcomes [113-115]. Aggressive management of haemodynamics is important, as is adequate pain management. In patients with ECG abnormalities and dyspnoea, additional diagnostics should be done to look for possible pulmonary oedema and/or cardiac failure. As LVD can be seen early on without any clinical symptoms of heart failure, yet echocardiographic examination is important for diagnosing NSM. When significant LVD exists, supportive inotropic medications should be considered. Although in an early study, treatment of LVD with dobutamine has some good effects, it is not the inotropic agent of choice for patients with SAH and TTS. This is because dobutamine is an adrenergic receptor agonist and can potentially exacerbate the syndrome [17, 111].

To treat cardiogenic shock and low cardiac output syndrome in these patients, a combination of inotropic and vasodilating medications can be used. A recent Cochrane review found no difference in mortality when using different inotropic medications. There is no evidence that a particular inotropic or vasodilating therapy is associated with any benefit regarding mortality [116].

Close monitoring of fluid balance should be initiated to avoid negative fluid balances. This could aggravate complications. In a study by Hoff et al. [111], SAH patients who developed vasospasm had significantly lower circulating blood volume than SAH patients without this complication. Close haemodynamic monitoring is also advocated as fluid-guided management and has been related to a better outcome [111].

## Conclusions

Of all neurological patients, SAH patients have the highest risk of developing NSM. SAH is recognised as the primary cause of TTS. The prognosis of NSM in SAH is not only related to the cardiomyopathy itself but also depends on underlying cerebrovascular complications. High prevalence of NSM in SAH patients warrants close monitoring of cardiovascular dysfunction.
